# Psychiatrists’ Illustration of Awe in Empathic Listening Assessments: A Pilot Study

**DOI:** 10.7759/cureus.55684

**Published:** 2024-03-06

**Authors:** Parameshwaran Ramakrishnan, Thomas M Brod, Thomas Lowder, Prasad R Padala

**Affiliations:** 1 Psychiatry, Baptist Health-University of Arkansas for Medical Sciences, Little Rock, USA; 2 Department of Theology, Ethics, and Natural Sciences, Graduate Theological Union-University of California at Berkeley, Berkeley, USA; 3 Semel Institute for Neuroscience and Human Behavior, University of California Los Angeles David Geffen School of Medicine, Los Angeles, USA; 4 Graduate Medical Education, Baptist Health-University of Arkansas for Medical Sciences, Little Rock, USA

**Keywords:** evidence-based medicine (ebm), metaphysics and phenomenology, analytical autoethnography, non-dual and non-agency, transpersonal mindfulness, transcendent unknowable, contemplative neuroscience, clinical chaplaincy, empathic listening, awe and non-agency

## Abstract

Background

“Awe” is typically an inspiring emotional response to perceptually vast stimuli signifying the transcendence beyond all cognitive frames of reference when we encounter the unexpected. Physicians’ experience of awe in clinical care interactions has not been studied in an empirical, evidence-based way. We aim to present a focused study of awe in a psychiatrist’s empathic listening (EL) assessments and propose an evidence-based framework to study it.

Methodology

This is an exploratory case series of a psychiatrist’s EL interactions (mean duration/*x̄* of 46.17 minutes) with six patients (two males and four females) aged 32-72 years (*x̄ *=54.67, σ = 16.64). Using the method of autoethnography, the verbal and nonverbal aspects of the EL assessments were analyzed and open-coded to generate qualitative data.

Results

The study revealed that the data in all the case studies could be classed into two thematic groups, namely, mindfulness and transpersonal mindfulness. The emotions of “awe” and “non-agency” were ubiquitous in all six case studies both for the psychiatrist and patients.

Conclusions

Recognizing the awe and non-agency in EL interaction is essential in conceptualizing the “mindfulness-to-transcendence” framework and the first step toward the evidence-based study of transcendence/metaphysics in phenomenological psychiatry.

## Introduction

Physicians have reportedly felt amazed at witnessing unexpected healing outcomes of their patients through their empathic listening (EL) interactions [1]. Such amazement in clinical healing outcomes has been identified as “awe” by physicians as well as patients [2]. Because of the lack of a conceptual understanding of how their patients could have felt healed in their EL process, physicians have self-described them as “magical” [3,4]. In clinical care, those “magical” EL moments occur unannounced and in uncharted ways amid the routine or mundane actions that clinicians perform, such as recording height and weight, assessing blood pressure, prescribing medications, and asking patients to complete validated questionnaires [3]. While witnessing sheer happiness in their patients, physicians may say, “I don’t know why; I didn’t do anything” (i.e., a feeling of “non-agency”) and the patient may merely say “Well. She listened” [3]. The word “listening” in this context refers to the psychiatrist/physician’s ability to empathize deeply with the feelings of pain and suffering that underlie the patient’s spoken and unspoken words. In this sense, the physician’s “empathic listening” is his/her “presence” or “just being there” with the patient’s pain and suffering [4,5].

Contrasting the lack of EL training in medical school or residency education, the clinical chaplains are trained [6] to intentionally use EL as their primary clinical care tool for transmitting and transmuting “awe” [7]. Clinical chaplains may refer to their process of EL and being present to patients’ pain and suffering as the “companioning the bereaved” model of care [8]. In their EL care, chaplains too have reported feelings of “providing nothing” (i.e., a feeling of “non-agency”) to their patients though they witness amazing healing outcomes among their patients [9]. Thus, the feelings of awe and non-agency may be ubiquitous in EL-based clinical care interactions.

Currently, awe is also described as an “emotional response to perceptually vast stimuli that transcend current frames of reference” [10], and a sign of a cognitive-schema-free state of mind [11]. Researchers have identified neural representation/correlates of awe [12,13], and of non-agency, a (nonpathological) apathetic state in which one is free from the conceptual thought of being a doer/doing [14]. Though these emotions are widely experienced by physicians and therapists [1,2], evidence-based studies are limited. While physicians have described the awe-inspiring healing outcomes as “magical,” clinical chaplains understand awe as an experience of the “Divine presence” in their EL care [9,15,16]. As physicians educated and trained in analytical thinking, the onus is on us to conceptualize the framework of automatic transcendence into the “thought-free” “cognitive schema-free” or “self-less” state of mind that is brought through the EL process. In this study, we will use the term Transcendence as a proxy for the term “Divine” [9] to avoid the need to indulge in theological discussions, which is not the focus of this study.

This paper aims to present a systematic assessment of a psychiatrist’s emotional experience of awe and non-agency on witnessing the patients’ healing transformation during EL interactions with six patients. The objective is to understand how the emotions of awe, and non-agency, which are described to have neural correlates can fit into the current contemplative neuroscience/mindfulness-based framework of EL assessment [17-19] and help in advancing evidence-based studies.

We hypothesize that the emotions of awe and non-agency signify the transcendental (beyond mindfulness) state of mind attained automatically through the EL process, a mindful meditative approach to psychiatric assessment [17-19]. In that sense, the experiential and neuroscientific understandings of the transcendental state of mind signified by the emotions of “awe” and non-agency can be built into the current mindfulness-based framework of the EL assessment process.

## Materials and methods

The study and its setting

This is an exploratory, retrospective study of six clinical case studies that met our inclusion and exclusion criteria. Three of the case studies were drawn from in-patient clinical chaplaincy settings at a tertiary care center in California. The other three case studies were drawn from psychiatric settings at a tertiary care medical center in Arkansas. Hospital guidelines were followed to eliminate the patient/participants’ identifiable privacy information. Inclusion criteria were patients over 18 years of age and older with a clinical diagnosis of mental disorder, and first contact with this researcher, RP. Minors (<18 years) may become suggestible during the EL interactions or resist the self-reflective or mindful-meditative process that EL interactions are supposed to facilitate. Hence, they were not included. Exclusion criteria were patients with acute psychosis and severe agitation, substance-related disorder in acutely intoxicated states, and any acutely ill neurological conditions, such as strokes and delirium. The University’s Institutional Review Board (IRB) was consulted, and the study was cleared as “not human subject research” and exempted from IRB approval.

Participants and materials

The first author of this paper (RP) is a psychiatrist who was also trained as a Clinical Chaplain in an Association of Clinical Pastoral Education (ACPE)-accredited Clinical Pastoral Education (CPE)/Spiritual Care residency program [6]. Clinical chaplains are trained in the art of EL, which is their primary assessment tool in the spiritual care process [17-19]. In this study, RP illustrates how EL care can be applied to psychiatric assessment for a better clinical outcome. The following are the summaries of the EL interactions between RP and his patients. The demographics, clinical care settings, duration of clinical interaction, and awe experience are listed in Table 1. The patients were aged between 32-72 years (mean age, *x̄* = 54.67 years and SD/σ = 16.64), and the mean duration of clinical assessment was 46.17 minutes.

**Table 1:** Participants’ demographics and their empathic listening assessment outcomes. MDD: major depressive disorder; PTSD: post-traumatic stress disorder; GAD: generalized anxiety disorder

Participant	Age (years)/Gender	Diagnosis	Visit duration	Hospital setting	EL assessment outcomes: -Psychiatrist’s unawareness/non-agency in patient’s healing (before patient’s own revelation). -Awe experience after witnessing the patient’s healing outcome and feedback
Patient 1	70/Male	MDD	45 minutes	In-patient	Non-agency: “Wow! How did the patient feel healed! I did not provide any substantial care to this patient!” Awe experience: “I woke up with my feet 6 feet above the ground.” “This is my real doctor”
Patient 2	68/Male	PTSD, MDD	60 minutes	In-patient	Non-agency: “What could I have done better to feet healed? Awe experience: The patient broke down into tears, revealed goosebumps, and reported a calming effect while describing the visit as “Spiritual” and “dazzling.”
Patient 3	32/Female	Schizophrenia	22 minutes	In-patient	Non-agency/Unaware: “What did I do for this profound rapport?” Awe experience: addressing the psychiatrist/RP as “Hello Jesus.”
Patient 4	34/Female	GAD, MDD	60 minutes	Out-patient	Non-agency: Unaware of the patient’s healing transformation. Awe experience: Witnessing sheer happiness in the patient
Patient 5	52/Female	MDD	60 minutes	Out-patient	Non-agency: Unaware of patient’s healing transformation. Awe experience: “My mother said, why are you going to a psychiatrist? Go and talk to Jesus about your troubles.” “I think I have come to the right place.”
Patient-6	72/Female	PTSD, MDD	30 minutes	In-patient	Non-agency: Unawareness of patient’s healing transformation. Awe experience: “Ah! that felt like God’s touch.”

Case 1

A 70-year-old Caucasian male, a retired physician, had a history of major depressive disorder (MDD) and non-small-cell lung cancer (Stage III), status post-right upper lung lobe resection five years ago. His depression was treated with 10 mg of escitalopram daily. The chaplain/RP-patient encounter occurred on the fourth day of the patient’s hospitalization. He was admitted for persistent pneumonia in light of an early (Stage I) recurrence of lung cancer. The patient shared his pain and regret for smoking cigarettes for several decades, his pain and guilt that his daughter died of cancer from secondhand smoke, and his current fear that the “*chemotherapy is not working*.” At the end of a 45-minute EL interaction, the patient became tearful, requested a prayer, and ended the visit. After leaving the patient, RP started to feel that “*I did not do anything substantive to help the patient*.” RP felt that a good cognitive behavior therapy session would be more helpful than EL for the patient and decided to follow up. The next morning, on a follow-up visit (15 minutes), the patient eagerly waved his hand as soon as he saw RP entering the floor, introduced RP to his nephew, profusely thanked RP for the visit on the previous night, and said: “*I woke up with my feet above the ground*.” His nurse informed him that the patient had eaten his breakfast for the first time in four days. When RP was leaving the room, he overheard the patient telling his nephew “*He is my real doctor*” (the patient was unaware that RP was a psychiatrist). RP felt pleasantly shocked, “*Wow! How did the patient feel healed? I thought I did not provide any substantive care to this patient!*”

Case 2

A 68-year-old Caucasian male with a history of post-traumatic stress disorder (PTSD) and MDD was grieving the recent demise of his 72-year-old partner. His symptoms were treated with 20 mg of citalopram daily. RP was called for spiritual care services. RP identified the major conflict the patient was going through was honoring his partner’s wish for his body to be donated to the anatomy department and cremated. The patient’s four family members who were sitting around the bed with the dead partner requested RP to say a prayer. Before settling down to pray, RP facilitated each of the family members to share their memories and stories with the deceased person. During the prayer, RP lifted all the memories that each of the family members had shared. By the end of the prayer, every family member, except this patient, was in tears, indicating how they felt touched by the empathic and healing presence of their chaplain/RP. After completing the visit, this chaplain left wondering “*What could I have done better to help the patient feel healed*,” as that patient was not in tears like the rest of the family. However, RP later discovered that the patient remembered RP’s presence as “*Spiritual*” and “*dazzling*” (see Appendices for the supervisor’s hand-written note describing the patient’s experiences). On receiving the note and the supervisor’s feedback that the patient had revealed the goosebumps that he was still experiencing, and broke down into tears, RP was pleasantly surprised, “*Wow! How did I not know about the patient’s healing!*”

Case 3

A 32-year-old African American female with schizophrenia was admitted to the closed mental health unit (MHU) with a relapse of her paranoia and disorganized behavior. She was mute and withdrawn. The chaplain/RP was called (on a Friday) by the unit’s nurse supervisor to visit with the patient to just “smile and say a prayer” as the patient was seen holding on to her Bible and not interacting with any staff. EL process was attempted despite the patient remaining mute or answering in monosyllables. Through his EL process, RP too stayed silent, being mindful of his thoughts and emotions, and reflecting on his frustrations, current and past. He reflected on his past frustrations with catatonic patients. Becoming mindful of his ruminations, he made a self-reflective statement, “*when we are preoccupied with our pain, frustrations, and struggles, we stay silent and not interact with people*.” The patient became mindful with such a self-reflective statement, and she shared her fear/paranoia, though very minimally. Staying mindful of her transference and setting aside his discomfort, the chaplain offered a prayer and ended the visit, feeling embarrassed with the care he provided for the patient. On the third day, Monday, when he was consulted for yet another psychiatric patient, he entered the MHU, still feeling embarrassed of his previous visit with patient 3 and hoping that he would not come face-to-face with her. However, as soon as he entered the MHU, this patient who was standing at the other end of the long corridor caught his eyes, waved her hand, and loudly addressed him saying “*Hello Jesus*.” The chaplain felt shocked to hear being addressed in such a profound way. “*Wow! What did I do for her to call me by that glorious name?!*” was the chaplain-RP’s question.

Case 4

A 34-year-old Caucasian woman with a history of general anxiety disorder and MDD came to the clinic with worsening anxiety symptoms. During the session, the psychiatrist/RP maintained reflective questioning, facilitating the patient to share her stories. While elaborating on her depressing stories, she started to reveal her difficulties in her current relationship. As she paused a bit, the psychiatrist paused his note-taking and turned toward the patient and noticed her voice was choking with pain. At the end of that interaction, the patient cheered up. The psychiatrist turned back with a pleasant smile that revealed his surprise, a moment of Awe! The patient, who was looking straight and deeply into his eyes said, “*Thank you for listening*.”

Case 5

A 52-year-old Caucasian woman being treated for chronic insomnia was referred by her primary care physician as she was not comfortable prescribing Zolpidem anymore. The patient shared her troubles with chronic insomnia and that she needed Zolpidem. As the psychiatrist listened (EL) to her stories, she started to pour over the details of sadness in her life, and her symptoms met the diagnosis of recurrent MDD that had never been addressed. After becoming tearful over and again sharing her stories with her EL psychiatrist/RP, she looked deeply into the psychiatrist’s eyes and revealed “*When I was leaving home to come to this clinic, my mother said, why are you going to a psychiatrist? Go and talk to Jesus about your troubles*.” She paused and added, “*I think I have come to the right place*.” The psychiatrist did not expect that coming and had a “Wow” moment then. After a long discussion, she agreed to start taking mirtazapine for her sleep, depression, and appetite.

Case 6

A 72-year-old woman with a history of PTSD and recurrent MDD was admitted to the medical floor for treatment of her chronic obstructive lung disease. Psychiatry was consulted for depression and passive suicidal thoughts. On this psychiatrist/RP’s assessment, she shared symptoms that amounted to current MDD with a moderate degree of severity. She also kept returning to share her past traumas. On EL to her references to past PTSD, she became tearful and shared her ongoing trauma from her current boyfriend. Touched by the empathy received during the session she broke down crying. Her hands became tremulous, and she held the railings of her bed. The psychiatrist gently placed his palm on her hand which was holding the railing. She calmed down, opened her eyes with a pleasant smile, and said: “*Wow! That felt like God’s touch*.” Such an expression was unexpected, and an Awe-provoking moment for this psychiatrist. The patient paused and said, “*Thanks for not judging me*.”

The focus of study in each of the above case summaries is on (1) how, through the psychiatrist’s EL interactions, the patients had experienced healing in the form of their increased rapport, vulnerable tears, goosebumps, cheerful affect, and religious/spiritual emotions. Such changes in mood and affect, even temporarily, are considered signs of healing [20-22]. (2) How those healing transformations were unexpected by the psychiatrist-chaplain (RP), and how the awe moments transpire in him. Thus, RP studied his mental-emotional processes that transpired when he interacted with each of the patients included in this study. In that sense, he is a participant-researcher, and hence a study material himself. Such a methodology of studying one’s own mental-emotional self while studying the patients’ healing experiences is elaborated below.

Methodology

Evidence-based study of awe in EL clinical interaction is an inductive process. In that, we start with the results and go backward to inductively analyze the qualitative data drawn from the EL interaction process. As seen in the case summaries (Table 1), the patients’ healing expressions came in unexpected ways at the end of the EL interactions provoking awe in RP. While the patients were crediting RP for their healing, RP was wondering “*How did that healing happen*!” “*Why is he crediting me*?” “*I did not provide any substantial care to this patien*t!” Such a feeling of having no role in patients’ healing, termed “non-agency” in this paper, has been reported by other EL physicians and chaplains as well [3,9]. These experiences of witnessing unexpected healing outcomes and the emotions of awe and non-agency become the signpost of evidence-based study of EL assessment.

The Mindfulness-Based Autoethnography Method of Studying Awe in EL Assessment

The study of one’s own behavior (including their thoughts and emotions) as they interact with people around them is called autoethnography, while the study of other individuals in an environment is ethnography [23]. A combination of methods of autoethnography and ethnography have been applied to study clinical chaplains’ EL/spiritual care assessments [24,25]. The autoethnographic study of clinical EL interactions includes two processes: First, the researcher/RP has to mindfully “zoom in” to observe the granular (“microscopic”) details of the subjective elements of own thoughts, feelings, intentions, urges, etc. These mindful observations of one’s intrapsychic world provoke self-empathy. Second, using wisdom and self-empathy, the researcher/RP would “zoom out” to mindfully observe how he interacts with the external world, the patient, and the clinical situation [17-19,26]. Thus, RP is a participant-researcher in this study.

Immediately after each patient visit, the EL care interactions were transcribed verbatim by RP using a contemporaneous note-taking methodology [17,18,24]. The granular details that are jotted down are of this psychiatrist/RP’s self-reflective observations of his and his patient’s speech and behavior, as well as his own intrapsychic processes, such as his thoughts, emotions, internal struggles, past painful memories, and urges that got triggered while listening to the patients’ stories [17-19,24,25].

The transcription itself is a mindful-meditative/self-reflective process, which includes (1) the psychiatrist’s mindful awareness of his thoughts and emotions that were getting triggered by the patient’s stories, (2) his struggles in staying non-judgmentally and empathetically present to the patient’s pain and struggles. Thus, the human “self” is the primary instrument of study in autoethnographic research [27]. Having a mindfulness-based framework of autoethnography is argued to lend itself to evidence-based contemplative neuroscientific studies [17,18]. Identifying the Aha/Wow, awe-inspiring moments that signify the transcendental mental state that transpires during their mindful-meditative process of the EL interaction is the key (signpost) to evidence-based studies on EL.

Open-Coding Method of Developing Qualitative Data for Inductive Analysis

EL interactions are methodical. To study these interactions, one needs to quantify the qualitative data. The qualitative data in the EL interaction includes the observable events such as the spoken and unspoken statements, facial expressions, body language, and physical interactions. In addition, because of the auto-analytical nature of its study, the qualitative data includes the metadata about the data provider/RP’s intrapsychic processes with which the qualitative data itself is produced. Thus, an autoethnographic study of the EL interaction is an epistemological process in which the participant-researchers would study/understand their own intrapsychic (thoughts and feelings) processes that are generating the data. The methodology of open coding for analyzing the qualitative data and the metadata to quantify them is described as epistemic network analysis (ENA) [28], and it has both human and machine-readable standards. The open-coding process, described in the past as grounded theory methodology [29], was typically based on human-readable standards, and it has been well-tested in studying EL assessments in the past [17-19]. Open coding enables us to study the seemingly disparate data sets amenable to be inductively analyzed and organized into a meaningful theoretical framework [30]. The open-coded qualitative data sets in this study were curated by human-readable standards that can be reproducible, enabling researchers to document their workflow, as well as organize their data in a format that is agnostic to software of any kind [28]. The open-coding method founded on human-readable standards is adopted and applied to code the qualitative data deductively and inductively. By deductive means the narrative corpus of the EL interaction was curated to produce three levels of abstraction: (1) verbatim statements of this researcher (RP) and his individual patients, including nonverbal expressions, such as silent pauses; (2) RP’s observations (O) as a participant-researcher; and (3) RP’s subjective (S) thoughts and feelings. The details of how these abstractions are developed into codable themes are presented in Table 2.

**Table 2:** Human-readable open-coding method of developing themes for analysis of empathic listening interactions.

Events	Description of codable events	The codes: Each event and related subevents are coded sequentially and uniquely, using letters and numbers
Observable speech	Verbal statements uttered by RP as the psychiatrist during his EL care assessment. Verbal and nonverbal statements (silent pauses) uttered by the patients (P)	Each statement of RP and his patient (P) are coded in sequence and assigned to the individual speaker as follows: coded as RP1, RP2, RP3, etc. Coded as P1, P2, P3, etc.
Observable behavior	These are RP’s observations (as the participant-researcher) of the patient and the self. The observations corresponding to each verbal interaction can be subtyped into (a) facial or emotional expressions, (b) body language, and (c) other behaviors	The observations (with the subtypes) of behaviors corresponding to each verbal interaction of the EL process are coded as follows: O1a, O1b, O1c, etc. O2a, O2b, O2c, etc.
Subjective thoughts and feelings	These are RP’s (self-reflective) subjective observations of his cognitive and emotive (intrapsychic) processes corresponding to each of the verbal/nonverbal interactions. They are subtyped into (a) those that refer to RP’s self, triggered by the patient’s statements or behavior; (b) how they informed RP about the patient; (c) what does RP do with those thoughts and emotions	These subjective observations with subtyped are coded as follows: S1a, S1b, S2c, S1d, etc. S2a, S2b, S2c, S2d, etc. S3a, S4b, S3c, S3d, etc. S4a, S4b, S4c, S4d, etc.

To illustrate how the EL interaction can be studied to identify the granular details that can be developed into themed abstractions that can be open-coded and analyzed, we are presenting the “Verbatim case report” of Case 1 (see Appendices).

## Results

**Figure 1:**
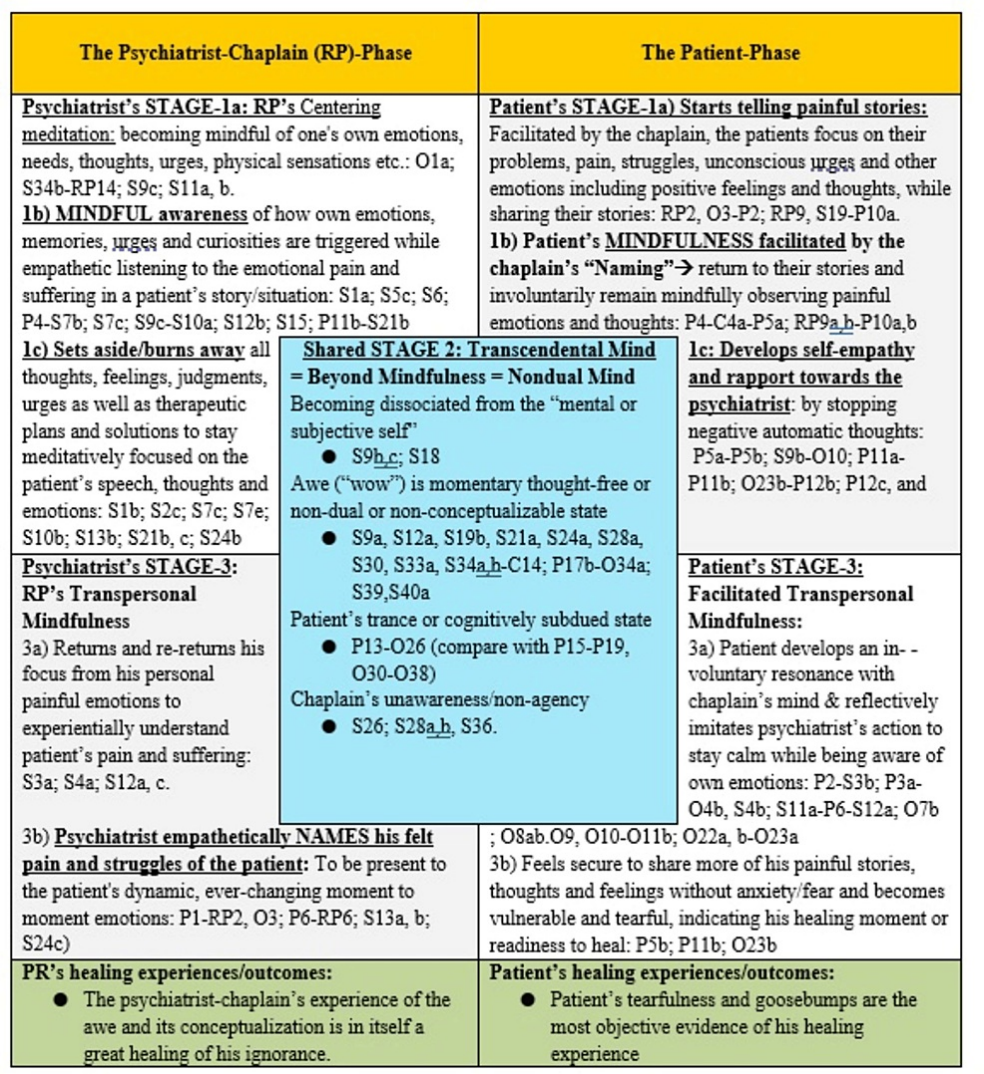
Tabular illustration of the mindfulness-to-transcendence framework of the empathic listening assessment process. The codes representing the verbatim statements of the first author (also the participant-researcher) RP and his patients and RP’s observations and subjective thoughts and feelings that got triggered in the empathic listening (EL) interaction have been elaborated in Tables 2, 3. After inductive analysis, those codes were assigned to the thematic groups of mindfulness, transcendence, and transpersonal-mindfulness, as indicated in this figurative table. Together, these thematic groups can be sequenced to form the “mindfulness-to-transcendence” (MT) framework, as discussed in the text. The contemplative neuroscientific underpinnings of this thematic MT framework were verified using electroencephalogram-based brain-computer-interface devices, which will be presented in the subsequent papers. Image credits: Ramakrishnan Parameshwaran.

The granular details which are open-coded (see “Verbatim report” of Case 1, Table 3 in Appendices) can be developed into the following thematic groups: (1) psychiatrist’s mindfulness, (2) psychiatrist’s transpersonal mindfulness, (3) patient’s facilitated mindfulness, and (4) patient’s facilitated transpersonal mindfulness, which were consistent with the findings observed in previous studies [17,18]. The phenomena of awe and non-agency were classed into the fifth theme of transcendence. To understand how the phenomena of transcendence could be organized into the mindfulness-based framework of the EL process, we further analyzed the granular details of the EL interaction that related to RP’s emotional experiences, awe and non-agency (Table 3 in Appendices, Figure 1).

Illustrating the phenomena of clinician’s non-agency in the patient’s healing

Please see Table 3 in Appendices and Figure 1 for the open codes that are referred to in this section. At the end of RP’s (45-minute) visit, the patient broke down into tears (O23b). Vulnerable tearfulness in therapy has been recognized to signify patients’ healing experience [17,18,20-22]. However, this patient did not verbalize any healing experience at that time. The patient’s thankful expressions at the end of that chaplaincy visit were subdued (P13-O26), contrasting his high elation the following morning (P17a, b; P15-19, O30-36, especially O33b). After his spiritual care visit, RP went about performing several routines such as entering his clinical notes, discussing the treatment plan with the nurse (O27a, b, c), and leaving the medical floor toward his night shift sleep room. Only after walking through the long hospital corridor, RP suddenly (S27a) started to feel *“I did not provide any substantial care to this patient*!” (S27a). It was as if he was in a deep meditative trance until that point on his walk through that long corridor. Such a state of trance or altered consciousness is reportedly experienced by hypnotherapists during their therapy sessions [31] and by meditators in their deep meditative states [32,33].

We argue that this psychiatrist-chaplain/RP had a similar experience of trance or altered state of consciousness from which he slowly emerged at the end of his long walk through the corridor several minutes after his EL session. This is similar to how hypnotherapists, for example, Gilligan, realize that they were in a trance while providing hypnotherapy to their patients only after reviewing the video recording of their hypnotherapy sessions [31].

The state of trance is intrinsic to a patient-therapist relationship, wherein both the therapist and the patient enter into a dissociative shared trance state [34]. Therefore, the patient may also have reflectively and unconsciously transcended into the selfless/beyond-mindful state along with RP and had experienced a similar trance state that may have corresponded with the time and duration of the trance experienced by RP. This trance-like state explains how the patient was subdued at the end of the EL/spiritual care session. In the case of RP, the deep meditative process which induces the trance state may also have freed him from the conceptual thought of being a doer/doing [14] and hence the feeling of “non-agency.”

Studying the phenomena of awe and mechanism of healing in EL/spiritual care

In a shared trance state [34] where the dichotomy of doer vs. doing and self vs. other is dissipated, the psychiatrist and the patient are said to be in a non-dual state of consciousness [14,35-37]. In this non-dual state is the perception of overwhelmingly expanded consciousness that transcended the individual’s (RP and patients’) restrictive cognitive schemas of self vs. other. In that transcendental state of mind, RP and the patient would have experienced the awe.

RP re-visited the patient expecting to find the patient in the same depressed state that he saw the previous evening (O27a, b). However, he was pleasantly surprised (S34a, RP14, see Table 3 in Appendices) upon witnessing the patient in a “happy” state of mind. That joy that followed the patient’s vulnerability and tearfulness (P5b, P11b, O23b) may be argued as his emotionally healed state, especially when we note how his vegetative functions such as appetite and sleep improved (P17b, O41a). RP’s verbal expression of “Wow!!” (RP14) and inner feeling (S34a) reveal his experience of awe, a pleasant surprise or shock from facing the unexpected outcome of the patient’s healing (S36). Furthermore, on closer study of the granular details of the EL interaction, one may note there were several occasions when RP experienced Awe/Wow (S9a, S12a, S19b, S21a, S24a, S28a, S30, S33a, S34a,b, S40a). These moments of Awe transpired as RP was shocked or surprised by unexpected disclosures of the patient (P5b, P10b, P11b, P12b) and a sudden turn of events in the interaction (P6).

To understand how RP could have possibly experienced shock or surprise on so many occasions during the EL interaction we pay attention to RP’s mindful-meditative activity of suspending all his judgmental thoughts and feelings (S7e, S8b, S10b, S11b, S13b, S20b, S23c). Such an act of suspending one’s judgmental thoughts (*epochē*, Greek) is understood as a religious reader’s meditative method of transcendence [38]. Thus, through the EL interaction, RP had repeatedly been transcending into the non-dual state of mind into which he continually invited the patient through mindful facilitation. As trance is intrinsic to such an empathic physician-patient relationship [34], the patient too would have experienced the transcendence and hence healing.

**Figure 2:**
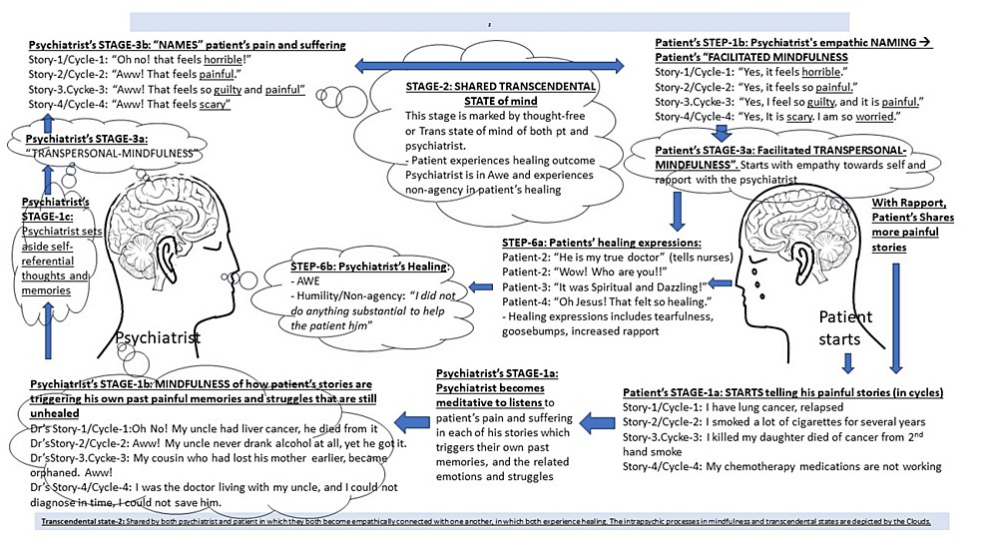
Figurative illustration of the mindfulness-to-transcendence framework of the empathic listening assessment process. Image credit: Ramakrishnan Parameshwaran.

Thus, the events of awe, non-agency, and the healing outcomes belong to the stage of “beyond mindfulness” or better termed “transcendence.” This stage of transcendence has not been reported previously in the literature discussing the chaplain’s EL process [17,18]. Accommodating this newly described state of transcendence into the mindfulness-based framework of chaplaincy is crucial for an evidence-based understanding of the transcendence aspects of the healing in the psychiatrist’s EL interactions. The mindfulness-to-transcendence (MT) framework of the EL assessment process is illustrated in Figure 1 and Figure 2.

## Discussion

Summary

In the six case studies presented in this paper, we have consistently found how an EL interaction is a mindful meditation process, which includes the following thematic stages: (1) the psychiatrist’s mindfulness. This stage begins with the psychiatrists’ empathic and attentive focus on the pain and suffering that the patients share verbally and nonverbally while telling stories about their losses. Then the psychiatrist would become mindful of how their painful memories similar to those of the patient’s loss and the related thoughts and emotions are getting triggered while listening to their patients’ stories. (2) While gaining an emotional and experiential understanding of the patient’s pain and struggles psychiatrists would continually discard all their self-referential and judgmental thoughts and feelings about their patients. In addition, they become indifferent/unattached to their own preconceived ideas, intentions, urges, and plans for treating the patient. In becoming indifferent to their clinical expectations and therapeutic ambitions (a positive and nonpathological approach), the psychiatrists gain the wisdom that the patients themselves have the innate ability to become self-empathic to heal themselves. Such wise psychiatrists would have entered the (3) transcendental or beyond-mindful state wherein, staying transpersonally mindful of their patient’s pain and struggles, they would merely verbalize to name the various emotions and struggles that their patients may be experiencing. (4) Such a “naming” process of wise psychiatrists would herald the patients’ stage of “facilitated mindfulness” in which the patients would become self-empathic to their pain and suffering related to their losses. Through the psychiatrists’ continued facilitation of their mindfulness, the patients enter their (5) stage of “facilitated transpersonal mindfulness,” in which they unconsciously and reflexively discard their mental and emotional preoccupations with their losses, reflecting their psychiatrists’ indifference to the idea of “fixing” their problems through cognitive schemas. Becoming free from their cognitive schemas is their self-transcendence. That transcendence is a conscious space that is beyond mindful awareness, which is shared by both psychiatrists and their patients. These thematic stages of the EL assessment process have been organized to arrive at the MT framework (Figures 1, 2).

In becoming free from all the cognitive schemas relating to their losses in life with which they were emotionally bound and stifled, the patients feel free from suffering. In that, they attain their empowered state of self-empathy and self-healing, though they would be in awe at their physician and credit the physician for their healing outcomes. Psychiatric textbooks do talk about how a psychiatrist’s diagnostic assessment, when conducted through EL, can be therapeutic and increase the patient’s sense of agency [39,40]. Psychiatrists, on the other hand, who had relinquished their therapeutic ambitions and intentions through the MT framework of the EL process, would naturally feel they have “not provided anything substantial to heal their patients.”

The study of the granular details of the subjective, mindfulness process is essential to understand how transcendence and its emotions of awe and non-agency emerge: the psychiatrist’s repetitive act of suspending all his cognitive processes (*epochē*) was essential for their self-transcendence. In that transcendent state, the psychiatrist had become child-like to be non-expectant of anything and therefore to be surprised by the patients’ disclosures and sudden changes in their speech and behavior and to be awed by them. On the part of the patients, with their growing trust in the psychiatrist, they would share more and more painful stories, as if peeling the onion, to reach the most painful story that may be troubling them in the current moment. Such self-revelation on the part of the patients is a sign of their growing rapport, which is, in turn, a sign of the loss of their self-preserving ego boundaries, which is their transcendence (Figures 1, 2). Elsewhere in this paper, we argued that this psychiatrist-chaplain/RP’s experience of trance or altered state of consciousness stretching beyond the EL care session was similar to the experience of hypnotherapists [31]. Thus, while not necessarily a focus of this paper, we have illustrated that the hypnotic state is achievable in an EL assessment without the psychiatrist establishing themself as a hypnotherapist.

Thus, in an EL interaction, “awe” is the emotion of the perception of vast consciousness (self-transcendent space) that transpires when the physicians and their patients become unbound by the cognitive schemas that differentiate them from one another. The deeply empathic presence, which in itself is a caregiving act, is indeed the act of a transcendental mind, which is an altered state of consciousness. In that state, psychiatrists become (positively or non-pathologically) apathetic to their therapeutic intentions and ambitions toward their patients. In turn, they are freed from the conceptual thought of having provided any substantial care to the patients, hence the feeling of non-agency in the patients’ healing outcome. Together, the emotions of awe and non-agency signify the physicians’ transcendence beyond their mindful awareness in which they function during the EL interactions.

This paper and its findings’ standing in the current scheme of things and future directions

Some of the mindfulness-based steps described in this EL process correspond to the Freudian principles of “ungrounded attention, unintentionality, and indifference (termed in this paper as ‘positive or nonpathological-apathy’) to therapeutic ambition” in psychoanalytic psychotherapy [41]. However, with our elaboration on how these Freudian principles could be closely related to the mindful and transcendental states of mind, which is the experience of awe, one may understand how Freudian psychoanalysis could have taken a turn toward a spiritual or transcendent position in Bionian supra-personal/spiritual psychoanalysis [42].

Recognizing awe and non-agency as emotions of transcendence is essential in conceptualizing the empirical MT framework of EL assessments, and the first step toward the evidence-based studies of it. To note, through this inductive analysis, we have closely studied neuroscientific literature on the emotions of awe and transcendence [10,11,35-37,43,44] to string the contemplative events of mindfulness, transcendence, and transpersonal mindfulness in the correct sequence to develop the MT framework of the EL assessment [17-19]. Going back and forth between the neuroscientific literature and the qualitative data confirmed that there could be a contemplative neuroscientific framework underpinning the empirical MT framework of the EL assessment process. We have also conducted follow-up studies to verify this MT framework using portable electroencephalogram (EEG)-based, brain-computer-interface devices, the findings of which will be presented as a sequel to this article.

While a transcendental state has been reported in meditation [45], we may be the first to illustrate how it is achievable by both the psychiatrist and their patient in a clinical setting through their EL (mindfulness-based meditative) interaction. It is through such a self-transcendent state that psychiatrists could grow deeply empathic to gain a first-person experience of their patients’ intrapsychic conflicts for improved accuracy in their diagnostic assessment; psychiatric textbooks do inform us of improved accuracy in EL assessments but they have not illustrated how [38,39]. Using the same case series studies, in the subsequent publication, we will illustrate how EL assessments in psychiatry could improve the diagnostic accuracy of psychiatrists.

Indeed, the self-transcending meditative state that is reported [43,44] as a self-healing state by other researchers is confirmed in this study. In that sense, even the psychiatrist would inevitably come out feeling healed as much as the patients do. This study illustrates and confirms how EL assessments could provide healing outcomes for psychiatrists and protect them from caregiver burnout, as mentioned in psychiatric textbooks [40]. This article illustrates how it is possible to study the emotions of awe and non-agency which signify the cognitive-schema free or the “unknowable” state of mind. In that, we agree with other researchers on the possibility of studying the unknowable [46,47]. Clinical chaplains who study their spiritual care (actually another term for EL) process refer to their scriptural texts and reverently ascribe the healing outcomes as the act of the Divine, the Unknowable Transcendent [9,15,16]. Discussions on how to understand the Transcendent using theistic language and constructs are out of the scope of this study but may be an inevitable next step if we have to integrate the disciplines of spirituality and psychiatry in an evidence-based way, for which there has been a growing demand [18,48,49].

Strengths and limitations

This is the first empirical study on the phenomena of awe and non-agency in EL assessment in psychiatry. In arriving at the MT framework, we have advanced the empirical framework of the EL assessment process which is grounded in contemplative neuroscience and hence amenable to application in evidence-based studies of it.

This researcher’s experiences of witnessing awe-inspiring healing outcomes may be argued as anecdotal. One may also argue that the healing expressions could be Kleinian manic-defense or “flight into health” [50,51] and hence a pseudo-healing. However, from our experiences in this case series, the limitations of the small sample size notwithstanding, we challenge those arguments because of the consistent findings across all six case studies. We also feel supported by similar publications on clinicians’ experiences of witnessing awe-inspiring healing outcomes in EL care [3,4,52]. We argue that replicability and reproducibility of the findings of this study can be possible by other psychiatrists after CPE training in EL assessment [6] or with training in the method of Bionian supra-personal psychoanalysis [41].

The kind of phenomena (awe, and non-agency) under consideration are those that cannot be observed or experienced by a second or third person. An attempt to reach objectivity was made by providing the supervisor’s hand-written note (see Appendices) which could serve as the third-person correlation grounded on the behavioral domain. An alternative to contemporaneous recording of the interaction after exiting the patient’s room could be an audio/video recording of the interaction itself. However, the subjective thoughts and feelings that the psychiatrist/EL provider experienced during the EL process can only be retrospectively re-read/re-heard from his memory. The accuracy of that recollection depends on the meditative state that the provider maintains during the contemporaneous note-writing process, which can be verified by continuing with the EEG-neurofeedback recording through the note-writing process. Though audio (better) video recording improves the objectivity of this kind of study, the classification of the EL process into focused attention, open mindfulness, and transcendental states (the MT framework) is possible only based on the subjective experiences of the EL care provider. How these subjective, contemplative states can be objectively verified using EEG frequency markers will be presented in our subsequent paper.

## Conclusions

This paper delves into psychiatrists’ experiences of awe and non-agency during their EL assessments to highlight them as signifiers of the transcendental state of mind. Through a qualitative and analytical approach, this research uncovers the nuances of the EL process, shedding light on the emotions of awe and non-agency that have previously been overlooked in clinical studies. By embracing a mindfulness-based research methodology, researchers can bridge the gap between scientific skepticism and the philosophical understanding of transcendental states, ultimately demystifying the healing mechanism behind EL assessments. With a deeper appreciation for the role of awe and non-agency in the healing process, psychiatrists can cultivate a pedagogy of EL assessments while still striving to understand the complexities of transcendental states. Despite the conceptual challenges that remain, this study of transcendence is essential for paving the way for future exploration of evidence-based studies on EL assessment in psychiatry and its therapeutic outcomes.

## Figures and Tables

**Figure 3:**
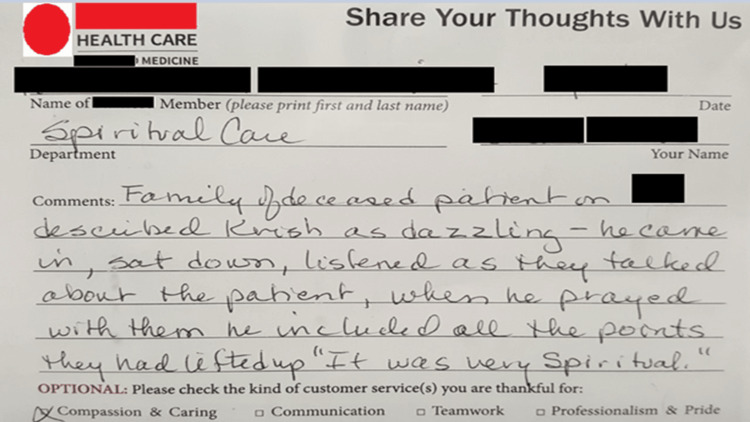
Chaplain-Supervisor’s hand-written note revealing the patient’s (Case 2) confession of his “dazzling” “spiritual” experience during the psychiatrist-chaplain, RP’s empathic listening assessment. Image source: Ramakrishnan Parameshwaran.

## Appendices

**Table 3:** Verbatin clinical case study 1 (truncated).

Verbatim of the chaplain/psychiatrist (RP) & patient’s (P) interaction	Objective findings: RP observation of the patient’s own expressions, body language, and other events during their interaction	Subjective findings: RP’s subjective (S) feelings, thoughts, urges, intentions, etc.
RP1a: Hi. I am a chaplain. My name is Krish. RP1b: Did you want to talk to a chaplain?	O1a: I entered the patient’s room after centering myself (meditating) for a few minutes. O1b: The patient was lying in bed. He appeared comfortable and was watching TV	S1a: He looks comfortable — what spiritual care he may be needing! I became curious. S1b: Several thoughts crossed my mind. I looked at them and set them aside to stay centered and focus on the patient
Table truncated here		
RP3: Yes, I know, and we can discuss it (Buddhism), but can I start by asking what brought you here to this hospital?	O6: The patient was lying in bed, alert and attentive toward me	S6: I was curious as to how he would respond to my initiation and about his current medical problems
P4: Chest pain. I had a lung tumor — the whole of this upper lobe was removed by surgery. The other lobes are expanding to cover the entire space of my chest	O7: He was gliding his arm over the left side of his chest — I mentally noted, “So it was a left lobe surgically removed.” O7b: He was looking at me most of the time, even as he was moving his hand over the left upper chest. My face reflected my painful feelings	S7a: I felt he was waiting for me to make an opening statement about his medical condition. S7b: Oh, it hurts. I remembered my own simple hernia repair surgery several years ago. And multiplied the intensity of pain and discomfort several times to understand how the patient must have hurt. S7c: I set aside my thoughts and memories and refocused on the patient’s story to realize that his problem is far more serious than mine — it is about the cancer that he mentioned. I felt sad. —truncated— S7e: I kept all my thoughts aside and decided to be in the present moment “It hurts”
RP4a: Aww! It hurts. … RP4b: (pause — did not know what to say further — I just kept quiet for a while) RP4c: … I am sorry you had to go through that	O8a: I empathize with his pain verbally. It is also reflected in my facial expressions. He was looking at me keenly. O8b: I could see some emotions on his face — “Is he anxious or worried?” I could not figure it out. I continued with my empathetic expressions and waited for him to say something. O8c: I paused	S8: I felt his pain and felt sad. I felt sorry for him and did not know what else I could say. But several curious thoughts crossed my mind: What is the need for the current admission? How serious is his current chest pain issue? Is it a relapse of lung carcinoma? I observed all my thoughts and realized that they were all my intellectual calculations meant to “fix” his problem. S8b: I kept aside my thoughts, waiting for the patient to share his
P5a: Yes, it hurts. … (paused). P5b: I smoked heavily for 50 years	O9: Started to look straight into my eyes. It was a stern look. The previous anxious/worried look had disappeared	S9a: Aww! I felt shocked (Wow) by his abrupt disclosure of his cigarette smoking habit. I had not yet made the automatic connection between his cancer with smoking. S9b: Is this his “confession” as if to a Catholic priest to atone for his mistakes and guilt? Is he feeling connected (rapport/trust)?—truncated—
RP5: Aww (silent pause)	O10: Aww, I sighed. My shoulders reactively slumped a bit. He looked at me with a questioning expression and a wonder, asking me, “How are you not judging me!?” I looked back straight at him with empathy and with my sadness. My eyes became a little wet, though I pulled back my tears	S10a: I remembered a similar expression on the face of an alcohol-dependent patient who was admitted for relapse and surprisingly asked me, “How are you not judging me?” Even in that situation, I had stayed without any verbal response, but my eyes had welled up. She had burst into tears merely by looking at my empathic sad eyes and expressions. S10b: I set aside all memories and returned to the current moment and stayed with my deeply empathetic sadness
Table truncated here		
P6: Do you watch this game?	O12: I felt stumbled—he changed the whole topic/stream of our conversation	S12a: Oh Wow! I felt shocked by his sudden shift in the conversation. I felt he was uncomfortable in staying with his pain, guilt, and other painful emotions. Did he also feel my pain and discomfort in counseling? —truncated—S12c: I decided to turn along with him to watch the TV show and wait for him to lead us back whenever he felt comfortable
Table truncated here		
RP10: Wow! You have six children	O20: I was cheerful to note that he has several children, but quickly, I noticed he was not happily telling me about his children	S20a: It felt good to hear he has a large family—so he may be having good family support, However, quickly, I withdrew from being happy as I noticed his unhappiness. S20b: I kept aside all my thoughts and feelings and focused on the patient
P11a: Yes. Only one of them lives here, with me—two are in Texas, one in Tennessee, and one in Florida … one daughter died 15 years ago … (long pause). P11b: She was just 40 years old, she died of cancer… (pause), and she did not smoke at all … (long pause)	O21a: His face appeared to turn serious and upset, but he did not say anything (There was a big, long silence) O21b: He stressed the word “died”—there was a lot of energy while saying that word—he stretched that word when he said it? O21c: He remained silent for a while	S21a: Wow! He said majorly painful stuff—it came as a big surprise, wow! and then the feeling of emotional pain dawned on me. I did not expect such a major painful issue from the demeanor that he presented. Several thoughts crossed my head: (1) Is he angry at his “God” because she died of cancer without smoking? (2) Is he feeling guilty for causing his daughter cancer from his secondhand smoke? S21b: I kept those thoughts away and felt some deep pain and sadness within and paid attention to those feelings in me. This patient’s situation was blatantly reminding me of the sudden death of my uncle, who died of liver cancer, and he was a teetotaler.—truncated—
RP11: Aww, that feels awful (pause) I am so sorry about that. (pause) It’s very painful	O22a: I was sad. My facial expressions and body posture gestures reflected my sadness. The patient turned towards me, O22b: The patient was visibly very sad. He continued to be silent. I, too, stayed silently	S22: I felt very sad for him. It’s a very painful feeling of losing someone very dear. I stayed silent, reminding myself of my pain of the past and feeling the pain that this patient may be going through. I remained present to that pain
(Silence)	O23a: There was a long moment of intense silence. He went back and forth, several times, looking at me and to himself with downcast eyes and again returning to look deeply at my face that was reflecting the sadness and some moisture in my eyes too. And then, O23b: He grimaced before bursting into tears. Till this point, he was holding it tight	S23a: I saw myself going back and forth between my pain from my uncle’s death and this patient’s grief in his daughter’s death. S23b: I felt him become deeply sad, and aww, a grown-up elderly man started to cry as a baby would—I felt it must be horrible - he could not hold on further. Aww. S23c: I kept aside all my self-referential thoughts and feelings and stayed feeling the patient’s pain
P12a: Yes … (pause) P12b: I am taking my chemotherapy, but it is not working … (pause) P12c: Will you pray for me!!	O24a: He is hopeless with the current medical treatments. O24b: He is very uncomfortable remaining with his painful stories in life. He wants to move away and end this visit by asking for prayer	S24a: Wow! I felt shocked by his revelation of failing chemotherapy. I felt sad. “Is he worried about his death from failed chemotherapy”? I had several questions to follow up on his “hopelessness” or about the “medication” that is not working. S24b: But I kept them all aside and stayed with the patient’s pain.—Truncated—
Table truncated here		
P13a: Thanks for your prayer. P13b: and thanks for coming	O26: He looked at me and bowed a bit after the prayer. I bowed back	S26: I was hoping he benefited from this visit
RP13: It was my honor to hear your stories and be a part of them. (truncated). Have a good night	O27: I nodded thankfully for having me visit him and waved back as he waved at me to say goodbye	S27: I left feeling very painful for him—remembering all that he said, he maintained a stoic image but was struggling so much, emotionally, and spiritually, and fragile from inside. What was he holding up for? Aww
P14: Sure. You are too	O28a: I walked out of his room. I stopped by the nurse’s station to write my notes and informed the patient’s nurse about his mental-emotional condition and that he may benefit from a detailed psychiatric assessment. O28b: The nurse said she too feels he is “depressed, he doesn’t talk much.” O28c: I stepped out of the floor and took the long corridor toward my night shift sleep room	S28a: Suddenly, (Wow!) After walking through the long hospital corridor and after a considerable time since leaving the patient’s room, I became very conscious of feeling that I did not do a good job with this patient. S28b: I came back to my night shift room and was thinking about him for a while. Did I provide him with enough care? I doubted myself, feeling “I did not do anything substantive to help the patient.” I need more time with him. Wanted to visit with him to follow up and provide better care. I thought of doing a good CBT session
	O29a: Early next morning, I visited the patient’s floor again, but I was hanging at the nurses’ desk—very hesitatingly. O29b: My back was facing the patient’s room. And then I heard the patient’s voice addressing me	S29: I was hesitating to meet him because I was unsure if he would want me to visit him again. Several thoughts were crossing my mind: Was this follow-up visit for my own need or the patient’s? I did suggest to him to ask for a follow-up, and he also sounded like that was a good suggestion, but I was not sure if he wanted to. I want to know how he was feeling after my previous night’s visit. I was searching for my reasons for follow-up. S29b: Several doubts and questions were rising in my mind, and I kept observing them and setting them aside even as I stood indecisively
P15: Hi Chaplain	O30: The patient shouted through his room door and across the nursing station. I turned and from a distance, I could see the patient waving his hand and calling out to me loudly	S30: I was pleasantly surprised. Wow! Very pleased to hear him call out to me. I felt at ease—the patient was indeed connected with me as much as was. So, both the parties have felt the need for a follow-up visit, I wondered!!?
	O33a: As I reached the room, I could also see his friend in full view—sitting at the foot end of the bed. O33b: The patient had a very broad smile. He extended his hand to shake hands with me. He leaned forward from his reclined position on his bed held my hand tightly and shook it vigorously as would a close friend do.—Truncated—	S33a: Wow! I could feel the energy in his handshake. I was pleasantly surprised. S33b: I appreciated the fondness with which he was introducing me to his friend
P17a: Thank you for stopping by last night. I felt so good. P17b: Wow! I am here now eating breakfast and woke up with my feet above the grass	O34a: I found the patient to be very happy and animated. There was glee on his face. His eyes were sparkling and wide. O34b: I smiled back with an equally pleasant face and glee	S34a: “Wow! He does feel good about my visit”—I felt very elated to see him happy. I was very pleasantly surprised by the stark difference in this patient’s mood and affect today as compared to the previous day. S34b: I did not understand the meaning of “feet above the grass” but it sounded as if “being elevated.” Wow! That was enough I did not ask him to elaborate on that. I kept my thoughts aside and became mindful of my feeling of awe
Table truncated here		
RP16: Nice, I will keep praying for you. You have a good visit with your friend	O39: I left the room and walked toward the nurse’s station to write my notes	S39: I was still wondering about ‘how this patient had benefited from my previous night’s visit in which I thought I did not do anything for the patient
P20: He is my real doctor	O40: I overheard the patient telling his friend as I was walking away from his room. [I had not mentioned that I am a physician nor there is a chance for him to know that from any other source because none of the staff knows that.]	S40a: Wow! I was super surprised. S40b: Is this how patients would feel when they have a good spiritual care visit?!! I felt that when a patient feels better emotionally then he/she feels good whether it actually changes the physical status of health or not—this is probably what is called “healing.” But how did it happen?
